# The Social Comparison Rumination Scale: Development, Psychometric Properties, and Associations With Perfectionism, Narcissism, Burnout, and Distress

**DOI:** 10.1177/07342829241238300

**Published:** 2024-03-09

**Authors:** Gordon L. Flett, Taryn Nepon, Paul L. Hewitt, Chang Su, Christa Yacyshyn, Kimberley Moore, Atieh Lahijanian

**Affiliations:** 1 7991 York University, Toronto, ON, Canada; 2 8166 University of British Columbia, Vancouver, BC, Canada; 3 1916 Brandon University, Brandon, MB, Canada

**Keywords:** social comparison, rumination, assessment, psychometrics, perfectionism, social media

## Abstract

In the current article, we describe the development and validation of the Social Comparison Rumination Scale. This measured was developed as a supplement to existing social comparison measures and to enable us to determine its potential relevance to perfectionism and other personality constructs. The Social Comparison Rumination Scale (SCRS) is a six-item inventory assessing the extent to which an individual is cognitively preoccupied and thinking repetitively about social comparison outcomes and information. Three studies with five samples of university students are described. Psychometric analyses established the SCRS consists of one factor assessed with high internal consistency and the measure is reliable and valid. Analyses showed that elevated levels of social comparison rumination are associated with trait perfectionism, perfectionistic automatic thoughts, perfectionistic self-presentation, ruminative brooding, burnout, depression, and fear of negative evaluation. Links were also established between social comparison rumination and both narcissism and dispositional envy. Overall, our findings support the further use of the SCRS and highlight the tendency of many people to think in deleterious ways about social comparisons long after the actual comparisons have taken place. We discuss social comparison rumination within the context of concerns about excessive social media use and young people being exposed to seemingly perfect lives that became a vexing cognitive preoccupation.

“I was depressed, really low. Hearing really great material by other groups made me feel as small as the dot over an I.”

Brian Wilson, after compulsively listening to the Beatles album Meet The Beatles

(Wilson & Gold, 1991, p. 90).

If comparisons are odious as Shakespeare warned us, the stench should be considerably worse for people who engage in excessive thinking and brooding about how other people are doing relative to them. The pressure and distress seem particularly acute for extreme perfectionists such as Brian Wilson who see standard setters as people who can keep raising the bar until it is out of reach for them. When these people compare with less capable people (i.e., downward comparison targets), they are believed to be seeking reassurance and checking on people they do not wish to become.

According to Festinger’s (1954) seminal theory, social comparison is a process of self-evaluation arising when individuals are uncertain about the adequacy of their performances or the correctness of their opinions. Social comparison involves using available information about other people as a guide for self-evaluation. Festinger’s (1954) seminal description of social comparison places a strong emphasis on the role of cognitive appraisal and mental evaluations and calculations of how an individual compares with other people in terms of performance and opinions.

We contend that for people who are heavily invested in social comparison and who have a chronically activated social comparison orientation, the cognitive side of social comparison extends to a cognitive preoccupation with social comparisons and dwelling on the outcomes of social comparisons. Given that unhappier people seem to be more strongly influenced by social comparison information (see Lyubomirsky, 2001), keeping comparisons going by dwelling on them should prolong and exacerbate unhappiness. If taken to extreme levels, social comparison rumination should prove highly deleterious in terms of potentiating and maintaining stress and distress and keeping the self cognitively activated. It can also set the stage for conditions such as burnout due to the cognitive exhaustion of a constant comparative focus and a sense of failing to keep up or to meet expectations. Exposure to negative social comparison information via rumination can also impair subsequent decision-making and the capacity to think (see Bandura & Jourden, 1991).

Social comparison rumination should be particularly problematic for young people who are excessively engaged in social media use. Research has provided us with numerous references to those young people who have frequent exposures to images of others with perfect lives (e.g., de Lenne et al., 2020; Devos et al., 2023; Weinstein, 2017). These images become worse if they are maintained in the minds of young people through the cognitive activation supplied by rumination. This exposure and related rumination should prove particularly problematic for perfectionistic young people with a social comparison orientation who are ego-invested in doing as well or better than their peers.

To our knowledge, a measure of social comparison rumination does not exist at present. However, the psychological literature contains several clues that point to the need for such a measure. Programmatic work on test anxiety, worry, and cognitive interference by Sarason and his associates incorporated a focus on thoughts about how others are doing (see Sarason, 1984; Sarason et al., 1996). For instance, the Cognitive Interference Questionnaire includes an item that inquires about thinking about how the other students were doing during a test situation (see Sarason et al., 1996). Clearly, such thoughts are salient among many students and those who become preoccupied with these thoughts can develop a form of rumination that can become a cognitively intrusive style that fuels worry and limits their ability to concentrate.

Our work in this area was inspired by a sage observation made two decades ago by Nolen-Hoeksema and her colleagues. Treynor et al. (2003) conducted a content analysis of what students ruminate about, and they emphasized that “… social comparison was a key aspect of the brooding factor in this study” (p. 257–258). This emphasis on social comparison thoughts is also reflected in the brooding scale item “Why do I have problems other people don’t have?” Nolen-Hoeksema (2003) further emphasized the social comparison theme in her book *Women Who Think Too Much*.

Social comparison rumination can result via upward comparisons with standard setters, but in other instances, it can be sparked by downward comparisons with people who typically do less well or are seen as having lower stature. This emphasis on social comparison rumination extends the conceptualization of the social comparison process. Reports of people ruminating about comparisons should make it evident that for many people, the act of engaging in social comparison does not end the process. In fact, it may escalate a person’s focus on social comparison.

The tendency to ruminate about social comparison information is especially important to consider when students are informed that they have done worse than other students or they suspect this is the case. Negative reactions that follow could reflect the distress caused by learning of the failure but also repetitive thoughts about not doing as well as others.

Our focus on social comparison rumination stems, in part, from seeking to identify mechanisms and processes contributing to the vulnerability of perfectionists such as Brian Wilson. An emphasis on social comparison has been discussed at length in analyses of perfectionism and its development, and an empirical link has been found between perfectionism and upward comparisons with standard setters (see Flett & Hewitt, 2022). Molnar and associates (2023) conducted a qualitative study with adolescent perfectionists and reported that social comparison emerged as a highly salient theme. This is significant because it underscores some perfectionists can competitively define success in terms of whether they outperform their peers and rivals.

One of the first indications of the role of social comparison and pursuing excessive standards came from classic work by Bandura and his colleagues. Bandura (1971) warned us of the potential costs of social comparison for people with exacting standards. Bandura noted that,

Many of the people who seek psychotherapy are behaviorally competent and free of debilitating anxiety, but they experience a great deal of personal distress stemming from excessively high standards of self-evaluation often supported by unfavorable comparisons with models noted for their extraordinary achievements (p. 31).

Similarly, David Burns (1980) described the distress of high achieving perfectionists who need to be “number one” and cannot handle being outperformed by very talented students.

Our primary reason for developing a measure of social comparison rumination is we reasoned that if social comparison is potentially destructive, especially among young people, then it is essential to identify facets of the construct that can account for how, when, and why social comparison can be deleterious. An emphasis on individual differences in social comparison rumination fits with a growing body of research that has confirmed that there is indeed a link between rumination and social comparison (e.g., Feinstein et al., 2013).

Festinger (1954) emphasized that social comparison will only occur when there are opinions and abilities that are important to the person. By extrapolation, we maintain that social comparison rumination will be most evident when outcomes and opinions are important and the stakes are higher. This rumination should be most evident when the person is in a state of ego activation and in need of validation. Social comparison rumination should also be particularly evident among people prone to envy.

The relatively brief measure of social comparison rumination described below was developed with item content not strongly laden with depression-related items. To our knowledge, the measure described in the current article is novel and is written in a format that can be modified for use with other populations. However, our current focus is on students. Why? There are myriad reasons that go beyond mere convenience. Vredenburg et al. (1993) noted that in addition to being at a stage in life involving greater uncertainty about the self and identity, university students experience considerable amounts of academic and social stress and “most college students are provided with a regular dose of evaluative feedback, and they also have countless opportunities to engage in social comparison.” This statement was written well-before students were able to access social media at any time of the day or night; social comparison has become even more possible and pervasive and students who travel down that road are arguably headed toward psychological landmines in terms of their self-evaluations.

Three studies with five samples are described below. Our overarching purpose was to evaluate a newly developed measure and establish its link with perfectionism. In addition, we sought to learn more about social comparison and potentially establish more of an emphasis on its cognitive aspects in the form of social comparison rumination.

## Study 1

Our initial study examined the psychometric properties of our new measure of social comparison rumination. It was anticipated that this new measure would be unidimensional and there would be clear individual differences in social comparison rumination that were assessed with an adequate degree of internal consistency.

Another purpose of Study 1 was to begin to evaluate the validity of this new measure by including measures of other constructs that should be associated with social comparison rumination. Our primary focus was on the link between social comparison rumination and measures of trait perfectionism and perfectionistic self-presentation. Flett et al. (2016) postulated as part of a cognitive approach to perfectionism that perfectionists are prone to engage in social comparison rumination as part of their emphasis on self-evaluative processes and as a reflection of the tendency for perfectionists to be hypercompetitive and in need of ego validation.

This study also included an assessment of dispositional envy. People characterized by dispositional envy tend to have elevated neuroticism and are prone to low self-esteem (see Smith et al., 1999). It is not difficult to envision an envious person who is consumed with obsessive thoughts about how other people are achieving and having a seemingly superior life. A link between dispositional envy and social comparison rumination would also be consistent with the observation from van de Ven and Zeelenberg (2020) that there are people who “… tend to ruminate more over the upward social comparison, making the experience more affectively negative and thereby increasing envy” (p. 237). If so, a link between social comparison rumination and dispositional envy should be clearly evident.

## Method

### Participants

Study 1 was based on two samples of participants. The first sample had 286 university students (223 women and 63 men). Their mean age was 20.4 years (*SD* = 3.5). The participants were recruited through the undergraduate research participant pool at a large Canadian university. They received credit toward their final grades in their introductory psychology courses in exchange for their participation. The majority of the participants (55.2%) were in their first year of university, with 24.1% in their second year. The most commonly reported intended majors were psychology (36%) and kinesiology (10.8%).

The second sample comprised 91 university students (67 women and 24 men). Their mean age was 23.6 years (*SD* = 9.3). They were recruited from the same institution and in the same manner.

### Procedure

First, participants in both samples provided their informed consent. They then completed a series of self-report measures in an online study. The following self-report measures were presented to the participants in the first sample in a randomized order.

#### Social Comparison Rumination Scale (SCRS)

The SCRS is a 6-item self-report questionnaire measuring brooding and rumination over social comparison outcomes in university students. The scale items were administered with five supplemental filler items that represent a general tendency to ruminate. Sample SCRS items include “I have been thinking a lot about how other students are doing versus how I am doing” and “Long after a test is over or a paper has been handed in, I keep thinking about how my performance compares with other students’ performance.” Items are rated on a scale ranging from 1 (*strongly disagree*) to 5 (*strongly agree*), with greater scores indicating greater social comparison rumination.

#### Multidimensional Perfectionism Scale (MPS; Hewitt & Flett, 1991)

The MPS is a 45-item measure assessing three trait dimensions: self-oriented perfectionism (e.g., “When I am working on something, I cannot relax until it is perfect”); other-oriented perfectionism (e.g., “If I ask someone to do something, I expect it to be done flawlessly”); and socially prescribed perfectionism (e.g., “The better I do, the better I am expected to do”). Extensive research attests to the reliability and validity of the MPS (Hewitt & Flett, 1991, 2004).

#### Perfectionistic Self-Presentation Scale (PSPS; Hewitt et al., 2003)

The PSPS is a 27-item measure assessing the need to appear perfect in public and defensively hide imperfections from others. It has three subscales: perfectionistic self-promotion (e.g., “If I seem perfect, others will see me more positively”); nondisplay of imperfection (e.g., “Errors are much worse if they are made in public rather than in private”); and nondisclosure of imperfection (e.g., “I should always keep my problems to myself”). The PSPS possesses good psychometric properties (Hewitt et al., 2003).

#### Brief Marlowe–Crowne Social Desirability Scale (Reynolds, 1982)

This 13-item true–false short form of the original Marlowe–Crowne Social Desirability Scale (Crowne & Marlowe, 1960) assesses the extent to which a respondent appears socially desirable. Sample items include “I sometimes feel resentful when I don’t get my way” and “No matter who I’m talking to, I’m always a good listener.” The psychometric properties of this brief version have been established (Reynolds, 1982; Zook & Sipps, 1985). This 13-item version is regarded as the best alternative to the original (see Zook & Sipps, 1985).

Participants in the second sample completed the SCRS and the following self-report measures in a randomized order.

#### Big Three Perfectionism Scale (BTPS; Smith et al., 2016)

This 45-item questionnaire measures three broad dimensions with three summary scores: Rigid perfectionism (e.g., “I strive to be as perfect as possible”); Self-Critical perfectionism (e.g., “I judge myself harshly when I don’t do something perfectly”); and Narcissistic perfectionism (e.g., “I am the absolute best at what I do”). Research has supported the reliability and validity of this scale (Smith et al., 2016).

#### Dispositional Envy Scale (DES; Smith et al., 1999)

This brief scale has eight items assessing an inclination toward envy. Sample items include “I feel envy every day” and “I am troubled by feelings of inadequacy.” Items are rated on a five-point scale with higher scores indicating higher dispositional envy. The *DES* possesses good psychometric properties (Smith et al., 1999).

## Results

### Descriptive Statistics

Table 1 presents the means and standard deviations for the total sample, as well as for men and women separately, on social comparison rumination for both samples. The alpha coefficient for the SCRS was .83 in both samples. Independent samples t-tests evaluated whether SCRS means were significantly different for men and women. No differences were obtained.

**Table 1.** Means and Standard Deviations of Social Comparison Rumination for Total Sample, Men, and Women.

	*Mean*	*SD*
*Study 1, Sample 1*		
Total sample	18.19	4.93
Men	17.97	4.40
Women	18.25	5.08
*Study 1, Sample 2*		
Total sample	18.12	5.46
Men	18.04	5.46
Women	18.15	5.50
*Study 2*		
Total sample	18.57	5.28
Men	17.59	4.98
Women	19.05	5.39
*Study 3, Sample 1*		
Total sample	17.54	4.52
Men	18.08	4.15
Women	17.33	4.65
*Study 3, Sample 2*		
Total sample	18.30	5.37
Men	18.09	5.80
Women	18.41	5.25

Note. *N* = 286 for Study 1, Sample 1; *N* = 91 for Study 1, Sample 2; *N* = 228 for Study 2; *N* = 134 for Study 3, Sample 1; *N* = 132 for Study 3, Sample 2.

### Confirmatory Factor Analysis

A confirmatory factor analysis (CFA) was conducted with the first sample using maximum likelihood estimation procedures to evaluate the model fit of the social comparison rumination scale. A one-factor model of social comparison rumination was tested with the seven items reflecting rumination over social comparisons. In order to assess model fit, the following indices were examined: Chi-square, Comparative Fit Index (CFI), Tucker–Lewis Index (TLI), Root Mean Square Error of Approximation (RMSEA), and the Standardized Root Mean Residual (SRMR). This model was an adequate fit, *χ*^2^ (14) = 62.144, *p* = .000, CFI = .931, TLI = .897, SRMR = .053, RMSEA = .110, 90% CI [.083, .138], and *p*_close_ = .000. All social comparison rumination items had factor loadings above .40. Although an alternative model with one removed was a slightly better fit, we retained this item because it is the only reverse-scored item of this scale. Instead, we removed another item because of its lower factor loading than the other items, and it was less face valid. The final model with six items is a better fit, *χ*^2^ (9) = 29.263, *p* = .001, CFI = .967, TLI = .945, SRMR = .037, RMSEA = .089, 90% CI [.054, .126], and *p*_close_ = .034. As can be seen in Table 2, all items had factor loadings of .42 or higher. The analyses reported above were based on the 6-item version.

**Table 2.** Factor Loadings for the Items of the Social Comparison Rumination Scale—Studies 1 and 2.

Items	Factor loadings	
	Study 1	Study 2
I have been thinking a lot about how other students are doing versus how I am doing	.71	.73
Long after a test is over or a paper has been handed in, I keep thinking about how my performance compares with other students’ performance	.75	.82
It is hard for me to stop thinking about how well my fellow students are doing	.83	.81
When someone does better than me, I spend a lot of time wondering why	.69	.74
It is pretty easy for me to stop thinking about how I am doing compared to how other students are doing (reversed)	.42	.21
Sometimes it has been hard for me to shut off thoughts about how well friends and acquaintances seem to be doing	.66	.78

Note. *N* = 286 for Study 1; *N* = 228 for Study 2.

### Correlational Analysis

For the first sample, the correlational analyses indicated that social comparison rumination was positively linked with all trait perfectionism dimensions: self-oriented perfectionism (*r* = .34, *p* < .001); other-oriented perfectionism (*r* = .12, *p* = .048); and socially prescribed perfectionism (*r* = .57, *p* < .001). Additionally, social comparison rumination was positively correlated with all perfectionistic self-presentation facets: perfectionistic self-promotion (*r* = .47, *p* < .001); nondisplay of imperfection (*r* = .55, *p* < .001); and nondisclosure of imperfection (*r* = .41, *p* < .001). Lastly, social comparison rumination had a negative, negligible association with the Brief Marlowe–Crowne Scale (*r* = −.14, *p* = .021).

Social comparison rumination in the second sample was positively correlated with all three BTPS summary scores: rigid perfectionism (*r* = .38, *p* < .001); self-critical perfectionism (*r* = .50, *p* < .001); and narcissistic perfectionism (*r* = .42, *p* < .001). Moreover, social comparison rumination was positively correlated with dispositional envy (*r* = .63, *p* < .001). To further underscore the links between trait perfectionism and reactions to how others are doing, note that dispositional envy was also correlated with scores on the Big 3 subscales: rigid perfectionism (*r* = .37, *p* < .001); self-critical perfectionism (*r =* .43, *p* < .001); and narcissistic perfectionism (*r* = .35, *p* < .001).

## Study 2

Our second study sought to replicate and re-assess the psychometric results found in Study 1. It was expected that this second study would yield additional evidence attesting to the SCRS as a brief and unidimensional scale with items that have adequate internal consistency. Several potential correlates of importance were also evaluated (e.g., depressive brooding and distress).

Second, in keeping with the links found between social comparison rumination and narcissistic perfectionism, we assessed two forms of pathological narcissism (i.e., narcissistic grandiosity and narcissistic vulnerability). Identification of a link between social comparison rumination and pathological narcissism would have significance in terms of illuminating a key self-evaluative mechanism that operates at the cognitive level to add to the distress and dysfunction of vulnerable narcissists. Narcissists with an overidealized view of themselves will be strongly impacted by others outperforming them or being more popular, and this can include a tendency to become obsessed and brood about these social comparisons.

Finally, this study evaluated a possible link between social comparison rumination and burnout in university students. Research has established that burnout is linked with having a social comparison orientation and a propensity to make upward comparisons with standard setters (see Buunk et al., 2001). This should generate considerable negative affect and a sense of a pressure to keep up with and perhaps surpass the performance and effortful striving of these standard setters; it follows that these tendencies will be magnified and be particularly deleterious among those students who tend to engage in frequent social comparison rumination.

## Method

### Participants

The sample had 228 university students (152 women, 75 men, and one person who did not report gender). Their mean age was 20.6 years (*SD* = 4.0). Participants were recruited through the undergraduate research participant pool in the same manner as in Study 1. Slightly more than half of the participants (52.2%) were in their first year of university, with 20.2% in their second year. Once again, the most frequently reported intended major was psychology (33.7%) followed by kinesiology (11.8%).

### Procedure

Questionnaires were administered to participants over the Internet following the same procedure as Study 1. Participants completed the SCRS and the following self-report measures:

#### Ruminative Responses Scale (RRS; Treynor et al., 2003)

We used the revised version of the original 22-item Ruminative Responses Scale (Nolen-Hoeksema et al., 1999), which consists of 10 items. Treynor et al. (2003) had re-analyzed data from Nolen-Hoeksema et al. (1999) and shortened it to 10 items. Participants are asked to “please read each item below and indicate whether you never, sometimes, often, or almost always think or do each one when you feel down, sad, or depressed.” The RRS is composed of reflection (5 items; e.g., “Write down what you are thinking and analyze it”) and brooding (5 items; e.g., “Think about a recent situation, wishing it had gone better”). Items are rated on a scale ranging from 1 *(Almost Never*) to 4 (*Almost Always*), with higher scores indicating ruminative responses to depression. Factor analyses support a two-factor model of rumination, and the subscales have good internal consistency and discriminant validity (Treynor et al., 2003).

#### Center for Epidemiological Studies-Depression Scale (Radloff, 1977)

The CES-D is a 20-item scale assessing the present level of depressive symptoms in the past week. This instrument measures both affective (e.g., “I felt lonely”) and somatic (e.g., “I had crying spells”) symptoms. Greater scores on this measure indicate a greater frequency of depressive symptoms. The CES-D possesses sufficient reliability and validity (Radloff, 1977).

#### Maslach Burnout Inventory – Student Survey (MBI–SS; Schaufeli et al., 2002)

The student version of the Maslach Burnout Inventory is a 15-item measure assessing 3 burnout facets: emotional exhaustion, cynicism, and personal accomplishment. Items are rated on a scale ranging from 0 (*Never*) to 6 (*Every day*), with higher scores reflecting higher burnout. We only used the emotional exhaustion (e.g., “I feel burned out from my studies”) and cynicism (e.g., “I have become more cynical about the potential usefulness of my studies”) subscales. Research has shown that the personal accomplishment subscale does not significantly load onto a burnout factor (Nepon et al., 2016; Schaufeli & Salanova, 2007).

#### Pathological Narcissism Inventory (PNI; Pincus et al., 2009)

The PNI is a 52-item measure assessing 7 dimensions of pathological narcissism: Entitlement Rage (e.g., “I get angry when criticized”); Exploitative (e.g., “I find it easy to manipulate people”); Grandiose Fantasy (e.g., “I often fantasize about performing heroic deeds”); Self-sacrificing Self-enhancement (e.g., “Sacrificing for others makes me the better person”); Contingent Self-esteem (e.g., “When people don’t notice me, I start to feel bad about myself”); Hiding the Self (e.g., “I often hide my needs for fear that others will see me as needy and dependent”); and Devaluing (e.g., “I sometimes feel ashamed about my expectations of others when they disappoint me”). There are two summary scores: Narcissistic Grandiosity (Entitlement Rage, Exploitative, Grandiose Fantasy, and Self-sacrificing Self-enhancement) and Narcissistic Vulnerability (Contingent Self-esteem, Hiding the Self, and Devaluing). The PNI possesses good psychometric properties in both student and clinical samples (Pincus et al., 2009).

## Results

### Descriptive Statistics

Table 1 displays the SCRS means and standard deviations for the total sample, as well as for men and women. The alpha coefficient for the SCRS was .84. An independent samples *t* test was performed to determine if the social comparison rumination means are significantly different in men and women; this *t* test was significant, *t* (158.27) = −2.02, *p* < .05. In this sample, women had a significantly higher level of social comparison rumination. However, the sample was composed of mostly women, so this finding must be interpreted with caution.

### Confirmatory Factor Analysis

A CFA was conducted using maximum likelihood estimation procedures to evaluate the model fit of the social comparison rumination scale. A latent social comparison rumination variable was composed of the six items reflecting rumination over social comparisons. Our model was an excellent fit, *χ*^2^ (9) = 17.937, *p* = .036, CFI = .985, TLI = .974, SRMR = .028, RMSEA = .066, 90% CI [.016, .111], and *p*_close_ = .241. All of the factor loadings are presented in Table 2. It should be noted that the reverse-scored item did have a low loading in this sample.

### Correlational Analyses

The correlational analyses revealed that social comparison rumination was positively associated with both brooding (*r* = .55, *p* < .001) and reflection (*r* = .34, *p* < .001). Social comparison rumination was also positively correlated with depression (*r* = .41, *p* < .001). The higher link with brooding was expected given that it is brooding that includes items such as the one that involves thinking about problems that other people don’t seem to experience.

Moreover, social comparison rumination was positively correlated with both narcissistic grandiosity (*r* = .45, *p* < .001) and narcissistic vulnerability (*r* = .58, *p* < .001). Regarding the student burnout subscales, social comparison rumination was linked with greater levels of emotional exhaustion (*r* = .38, *p* < .001) and cynicism (*r* = .42, *p* < .001). Lastly, brooding was positively correlated with depression (*r* = .57, *p* < .001).

#### Partial Correlation

A partial correlation analysis evaluated whether the link between social comparison rumination and depression is still significant after partialling out the effects of ruminative brooding. The results from this partial correlation indicated that social comparison rumination was still significantly and positively linked with depression, after controlling for brooding (*r* = .14, *p* = .031), though it is evident that the link was now considerably weaker.

## Study 3

Study 3 addressed another set of core issues. First, we evaluated the extent to which social comparison rumination is linked with the broad social comparison orientation that is believed to reflect a stable and dispositional personality orientation (see Gibbons & Buunk, 1999). We also tested the hypothesis that social comparison rumination is associated with a joint orientation toward upward and downward comparison.

Second, this study examined likely associations between social comparison rumination and elevated frequency of automatic thoughts about needing to perfect and needing to stop procrastinating and engaging in dilatory behavior. A cognitive preoccupation with social comparison could be a catalyst for additional thoughts involving perfectionism and procrastination, but students characterized by perfectionism and procrastination also have been known to have self-evaluative tendencies that are byproducts of a negative and uncertain sense of self (for a discussion, see Flett et al., 2004). Perfectionism and procrastination are both believed to reflect awareness of being discrepant from goals, ideals, and standards (see Smith et al., 2017), and it may similarly be the case that social comparison rumination results in a cognitive focus and heightened awareness of being someone who is falling short. A link with perfectionistic automatic thoughts can be seen as evidence of concurrent validity in that the Perfectionism Cognitions Inventory developed by Flett et al. (1998) included a few items with a social comparison focus (e.g., I have to be the best. My work has to be superior).

Finally, this study revisited the proposed link between social comparison rumination and poor adjustment. In this study, we measured depression and the fear of negative evaluation. An association with fear of negative evaluation would be in keeping with this type of fear being tied to insecurities and negative views of the self.

## Method

### Participants

Study 3 was based on two samples. The first sample comprised 134 university students (37 men and 97 women). Their mean age was 20.1 years (*SD* = 3.7). Participants were recruited in the same manner as in the previous studies. The majority of the participants (61.9%) were in their first year of study, and 27.6% in their second year. The most frequently reported intended majors were psychology (41.8%) and kinesiology (29.1%).

The second sample had 132 university students (34 men, 97 women, and one person who did not report gender). Their mean age was 20.1 years (*SD* = 2.8). Participants were recruited in the same way as the previous studies. The majority of the students (59.8%) were in their first year of university, with 18.9% in their second year, 11.4% in their third year, 3% in their fourth year, and 0.8% in their fifth year. The most commonly reported intended majors were psychology (31.1%) and kinesiology (19.7%).

### Procedure

After providing their informed consent, participants in the first sample were administered a series of self-report measures in an online study. In addition to the SCRS and CES-D, the following self-report measure was used:

#### Iowa-Netherlands Social Comparison Measure (Gibbons & Buunk, 1999)

The INCOM is an 11-item scale assessing individual differences in the frequency to engage in social comparison. For the current sample, we used the modifications recommended by Butzer and Kuiper (2006), which involved removing any frequency-related words (e.g., “often”) and using a different rating scale. Instead of respondents rating their agreement or disagreement with each item, they rated each item on a scale ranging from 1 (*never*) to 5 (*always*), with greater scores indicating a greater frequency of social comparison. Sample items include “I pay a lot of attention to how I do things compared with how others do things” and “I compare myself with others with respect to what I have accomplished in life.” The INCOM has good reliability and validity (Gibbons & Buunk, 1999). Moreover, the revised version has high reliability, with an alpha coefficient of .86 (Butzer & Kuiper, 2006).

#### Frequency of Upward and Downward Social Comparison (Butzer & Kuiper, 2006)

This 12-item scale was modified from the INCOM to measure with two 6-item scales the frequency of upward and downward social comparison. Upward social comparison involves making comparisons with individuals who are perceived to be better than oneself. Downward social comparison involves making comparisons with individuals who are perceived to be worse than oneself. An upward comparison item is, “When it comes to my personal life, I compare myself with others who have it better than I do.” A downward comparison item is, “I compare myself with others who have accomplished less in life than I have.” Items are rated on a scale ranging from 1 (*Never*) to 5 (*Always*). These subscales have high internal consistency (Butzer & Kuiper, 2006).

#### Perfectionism Cognitions Inventory (PCI; Flett et al., 1998)

The PCI is a 25-item scale assessing the frequency of perfectionistic automatic thoughts over the last week. Sample items include, “Why can’t things be perfect?” and “I should be perfect.” Items are rated on a scale ranging from 0 (*Not at all*) to 4 (*All the time*). Greater scores reflect a greater frequency of perfectionistic cognitions. The psychometric properties of the PCI are well-established (Flett et al., 1998, 2007).

The participants in our second sample completed a series of self-report questionnaires in an online study after providing their informed consent. These measures included the SCRS, the brooding subscale of the Ruminative Responses Scale, and the CES-D, which have already been described. The following self-report measures were also administered:

#### Procrastinatory Cognitions Inventory (Stainton et al., 2000)

This 18-item measure assesses the frequency of automatic thoughts related to procrastination. Representative items include, “No matter how much I try, I still put things off” and “I’m such a procrastinator, I’ll never reach my goals.” Items are rated on a scale ranging from 0 (*not at all*) to 4 (*all of the time*). Higher scores reflect a higher frequency of procrastinatory cognitions. This measure has good internal consistency, with alpha coefficients above .87 in three samples of students (Flett, et al, 2012). Its validity has also been established (Flett, et al, 2012).

#### Brief Fear of Negative Evaluation Scale (Leary, 1983)

The BFNE scale is composed of 12 items; however, it was later recommended that only 8 items be used without the reverse-coded items (Rodebaugh et al., 2004). Fear of negative appraisals of the self from other people is assessed by items such as “When I am talking to someone, I worry about what they may be thinking about me” and “I am afraid others will not approve of me.” Items are rated on a scale ranging from 1 (*Not at all characteristic of me*) to 5 (*Extremely characteristic of me*), with higher scores indicating a higher fear of negative evaluation. The BFNE scale seems as psychometrically sound as the original (see Leary, 1983). Further, Rodebaugh et al. (2004) found that the BFNE items had greater discriminant validity than the original scale and convergent validity was stronger for the straightforward items used in the current study.

## Results

### Descriptive Statistics

Table 1 shows the SCRS means and standard deviations for the total sample, as well as for men and women. The social comparison rumination scale had an alpha coefficient of .80 in Sample 1 and .85 in Sample 2. There were no significant gender differences in SCRS scores in either sample.

### Correlational Analyses

In Sample 1, social comparison rumination was positively linked with social comparison orientation (*r* = .40, *p* < .001), as well as with both upward social comparison (*r* = .24, *p* < .01) and downward social comparison (*r* = .39, *p* < .001).

Social comparison rumination was positively correlated with perfectionistic automatic thoughts (*r* = .47, *p* < .001). Social comparison rumination was also positively associated with depression (*r* = .18, *p* < .05). Additionally, both upward social comparison (*r* = .29, *p* < .001) and downward social comparison (*r* = .24, *p* < .01) were positively associated with depression. Social comparison orientation, however, was not significantly correlated with depression (*r* = .12, *p* = .183). Lastly, perfectionistic automatic thoughts were linked with greater levels of depression (*r* = .43, *p* < .001).

Table 3 presents the Sample 2 correlations. The results showed that social comparison rumination was positively correlated with brooding, procrastinatory cognitions, depression, and fear of negative evaluation. Brooding was positively associated with procrastinatory cognitions, depression, and fear of negative evaluation. Further, procrastinatory cognitions were associated with higher depression and fear of negative evaluation. Lastly, depression and fear of negative evaluation were positively correlated with each other.

**Table 3.** Correlations Among Social Comparison Rumination, Brooding, Procrastinatory Cognitions, Depression, and Fear of Negative Evaluation—Study 3, Sample 2.

	1	2	3	4	5
1. Social comparison rumination	–				
2. Brooding	.43**	–			
3. Procrastinatory cognitions	.33**	.50**	–		
4. Depression	.27**	.56**	.40**	–	
5. Fear of negative evaluation	.47**	.54**	.45**	.42**	–

Note. *N* = 132. **p < .01, two-tailed.

### Regression Analyses

For Sample 2, two multiple regression analyses were performed to determine which variables predict depression and fear of negative evaluation. Specifically, the following predictor variables were all entered simultaneously for both analyses: social comparison rumination, brooding, and procrastinatory cognitions. Before conducting these analyses, we checked for the normality of the outcome variables (i.e., depression and fear of negative evaluation) and the distributions differed from normal. Thus, the robust bootstrapping procedure was employed because it does not impose the normality assumption. We generated 5000 bootstrap samples to provide estimates, standard errors, and 95% bias-corrected confidence intervals.

For the regression predicting depression, social comparison rumination, brooding, and procrastinatory cognitions were all entered into the predictor block, and depression was entered as the outcome (see Table 4). This model significantly predicted 33.7% of the variance in depression scores, *F* (3, 128) = 21.67, *p* < .001. The only significant unique predictor of depression was brooding.

**Table 4.** Summary of Multiple Regressions for Variables Predicting Depression and Fear of Negative Evaluation—Study 3, Sample 2.

Variables	*R* ^2^	B	SE B	95% CI
*Predicting Depression*				
Step 1	.337***			
Social comparison rumination		.04	.16	[-.26, .31]
Brooding		1.34***	.27	[.75, 1.93]
Procrastinatory cognitions		.09	.06	[-.02, .20]
*Predicting Fear of Negative Evaluation*				
Step 1	.396***			
Social comparison rumination		.41**	.14	[.13, .67]
Brooding		.74***	.20	[.36, .1.11]
Procrastinatory cognitions		.10*	.04	[.02, .18]

Note. *N* = 132. *p < .05; **p < .01; ***p < .001.

For the regression predicting fear of negative evaluation, social comparison rumination, brooding, and procrastinatory cognitions were again entered into the predictor block, and fear of negative evaluation was entered as the outcome (see Table 4). This model significantly predicted 39.6% of the variance in fear of negative evaluation scores, *F* (3, 128) = 27.94, *p* < .001. Social comparison rumination, brooding, and procrastinatory cognitions were all significant individual predictors of fear of negative evaluation.

## General Discussion

The main goal of this research was to develop a brief measure of individual differences in social comparison rumination. Three studies involving multiple samples were described. Overall, this research attests to the presence of individual differences in social comparison rumination and the feasibility of assessing these differences. We developed a six-item one-factor measure with acceptable psychometric properties including an acceptable level of internal consistency. It was decided to retain one item that was worded negatively even though this yielded weaker results. Potential users of this instrument can decide whether they will retain this item.

We also found that this new measure had a negligible association with social desirability scores. Evidence of concurrent validity was obtained in terms of the numerous research findings linking social comparison rumination with other measures tapping ruminative thought and automatic thoughts.

The SCRS items were worded in a manner that emphasized repetitive and persistent thoughts about social comparison. This research confirmed that certain students have frequent thoughts about social comparison information and outcomes. As expected, social comparison rumination was associated with the broad social comparison orientation that is framed as a personality style (see Gibbons & Buunk, 1999). Findings from Study 3 established that social comparison rumination can result from comparing upward with standard setters but also from comparing downward with peers of lower stature and lower levels of achievement. People with extreme levels of social comparison rumination likely have engaged in upward and downward comparisons and in terms of their tendencies, they resemble the children described in Ruble and Flett (1988) who sought social comparison information across a broad range of comparison targets.

The studies described in the current article yielded extensive evidence that linked social comparison rumination to distress among students. Associations were found not only between social comparison rumination and depression but also between social comparison rumination and fear of negative evaluation. These associations were found concurrently, and it will be important to evaluate in longitudinal research the extent to which social comparison rumination contributes to vulnerability to the onset of distress and to the persistence of distress over time.

Our results confirmed that social comparison rumination is associated quite robustly with dispositional envy. It is likely the case that there is a rumination component that is specific to the experience of envy that likely parallels the more general association with social comparison rumination. An obsessive preoccupation with how others are doing can result in an ego-focused and envious performance orientation that seems likely to undermine well-being. As we noted in the introduction, this likely applies to certain young people who are heavily engaged on a daily basis with social media.

One overarching primary objective of this research project was to evaluate the link between perfectionism and social comparison rumination. We found extensive evidence suggesting that social comparison rumination is a key process that tends to add to the vulnerability of ego-involved perfectionists who are uncertain and likely high in self-doubt. The results of our initial studies confirmed that various elements of the perfectionism construct are associated with social comparison. Regarding elements of the comprehensive model of perfectionistic behavior (see Hewitt et al., 2017), trait perfectionism, perfectionistic automatic thoughts, and perfectionistic self-presentation were all associated with social comparison rumination. The link with more frequent perfectionistic automatic thoughts points to the likelihood that perfectionistic rumination and social comparison rumination are often interconnected among students who feel a strong need to be perfect and are aware of falling short of being perfect but are unable to stop thinking about their social comparison standing and their peers and competitors. As for the results involving trait perfectionism, given that socially prescribed perfectionism is often highly debilitating (see Flett et al., 2022), the association between this perfectionism dimension and social comparison rumination is potentially quite important; frequent social comparison rumination can provide frequent reminders of failing to keep up with peers and falling short of expectations and standards. Bandura (1971) noted that children in his research often patterned themselves after an adult who modeled working according to exacting standards. He proposed that failing to achieve at the same level is a recipe for self-devaluation and a paucity of self-reward. This harshness toward the self and dissatisfaction should be exacerbated among perfectionists who are cognitively preoccupied with social comparisons and associated outcomes.

The associations between social comparison rumination and the facets of perfectionistic self-presentation were particularly strong and are consistent with the insecure and negative sense of self that underscores trying to portray oneself as perfect and avoiding seeming imperfect in public (see Hewitt et al., 2003). It is likely that there are several factors and tendencies that underscore these associations, including people sensing that they need to emulate others in order to seem perfect and not be exposed as imperfect and unable to keep up with others. Unfortunately, if perfectionistic self-presenters are over-represented among young people who go online and portray themselves as perfect, they are suffering personally yet adding to the myth of the so-called “perfect life.”

Future research needs to consider social comparison rumination as a factor that mediates the associations known to be evident with perfectionistic self-presentation. For instance, frequent and cognitively salient social comparison rumination seems like a strong candidate for being a mediator of the link between perfectionistic self-presentation and feelings of being an imposter and related feelings of distress and fear of negative evaluation.

The findings with the BTPS linked social comparison rumination with self-critical perfectionism, narcissistic perfectionism, and rigid perfectionism. The strongest link was with self-critical perfectionism. This BTPS subscale includes brief subscales tapping socially prescribed perfectionism, self-criticism, doubts about actions, and concern over mistakes. The self-doubt and self-criticism of self-critical perfectionists likely increase their tendency to engage in social comparison rumination. However, this is one instance in which reciprocal associations likely come into play; it is not too difficult to envision a sequence in which ongoing social comparison rumination increases both the levels of doubts about actions and concerns over mistakes but also the perceived importance of comparing favorably with others going forward.

The link between social comparison rumination and narcissistic perfectionism found in Study 1 was supplemented by Study 2 results showing that pathological narcissism in terms of narcissistic vulnerability and grandiosity were both associated with social comparison rumination. Social comparison rumination may contribute to and sustain the insecure and uncertain sense of self that underscores narcissism. Perhaps this rumination fuels the hypercompetitive tendencies that can cause interpersonal problems for ego-invested narcissists seeking validation.

Other results confirmed that social comparison rumination was associated with ruminative brooding and a higher frequency of procrastination-related automatic thoughts. In addition, social comparison rumination was associated with depression and fear of negative evaluation. This research established in one sample that social comparison rumination did not predict unique variance in depression scores, but it did predict unique variance in fear of negative evaluation, beyond the variance attributable to ruminative brooding and the experience of frequent automatic thoughts about procrastination.

We found uniquely in Study 2 that social comparison rumination was associated significantly with burnout in students in terms of higher scores on the facets of cynicism and emotional exhaustion. We believe that this finding is potentially quite meaningful and is one that ideally results in future programmatic work. Social comparison rumination may be implicated here in various ways. Burnout may result from the disappointment and demoralization of not measuring up to others, but also excessive rumination with a comparative focus may also add to a state of cognitive exhaustion. The social comparisons and associated ruminations here can be with respect to performance outcomes, respective levels of effort and striving, or thoughts and observations tinged with envy about how other people seem to be having it easier in terms of workload and in terms of life.

While we have focused on the SCRS, it is essential to consider social comparison rumination from a broad perspective as a personality construct and a cognitive activity. Several themes and issues need to be explored. For instance, our participants were university students and the scale was worded to reflect its use with students. Individual differences in social comparison rumination should be evident among people in general, including adults in midlife. The wording of the scale items is appropriate for use with adolescents and perhaps children, but for adults, the wording can be modified slightly to make the Social Comparison Rumination Scale broadly applicable.

Flett et al. (2016) noted that social comparison rumination extends beyond specific achievement settings because it follows that people engage in social comparison rumination about their lives. As we have noted, this type of rumination may underscore the destructiveness of excessive social media, which represents a key context where social comparison is believed to take place. Social comparisons involving appraisals about how people’s lives are going are quite easy to make and the person who is ruminating about how their life is going versus how other people seem to be doing in their seemingly perfect lives is someone who is likely to experience significant psychological pain due to being acutely and chronically aware of life discrepancies.

It also seems essential to consider social comparison rumination from a situational perspective. What contexts are associated with more versus less social comparison rumination? Future research with a contextual focus can explore how personality dispositions interact with situational factors to impact levels of social comparison rumination. For instance, to what extent are people more likely to ruminate about comparisons in highly ego involving situations?

Efforts to understand social comparison rumination should embrace a broad approach. This type of rumination can be evaluated in terms of the frequency and intensity of social comparison thoughts, but social comparison rumination can also be examined in terms of its perceived antecedents and consequences as well as the experience of rumination itself in terms of the extent that it is seen as an intrusion or a distraction. A related line of inquiry can consider whether rumination about comparison makes it more or less likely that people will rely on self-protection mechanisms.

### Directions for Future Research

Social comparison is ubiquitous and, as such, there are countless other key directions for future research on individual differences in social comparison rumination. First, and foremost, more information is needed about the SCRS. Some key issues remain to be explored including the temporal stability of social comparison rumination. We regard this as a personality disposition and, as such, the SCRS should yield scores with significant temporal stability, but with the caveat that it should be the case that situational factors and life experiences will also elicit thoughts about rumination. Second, the SCRS and its characteristics need to be evaluated with participants from various cultures and in younger students. We have already begun this process with research on social comparison rumination in children and adolescents from China.

Social comparison rumination needs to be studied in various specific contexts and in specific domains. For instance, given the relevance of social comparison in evaluating various aspects of physical appearance, there is merit in exploring the development of a measure of appearance-related social comparison rumination. Similarly, given individual differences in social comparison orientation of ability and social media social comparison orientation (see Yang, 2022; Yang & Robinson, 2018), measures of rumination in these specific domains also seem essential.

Future research is also needed to more fully examine the nomological network of social comparison rumination. In addition to other personality correlates not included in our initial studies, such as self-consciousness and fear of missing out, more work is needed on beliefs and assumptions that should be linked with a propensity to engage in social comparison rumination. For instance, rumination should be more characteristic of people who have a fixed mindset who tend to see themselves and others in dispositional terms. The tendency to see ability differences as deeply ingrained and constant should also translate into greater social comparison rumination among those people who feel they are not keeping up with their peers. Future research should also examine the daily life experiences and emotional reactions of people with high versus low social comparison rumination.

In summary, in the current article, we described a brief new unidimensional measure of social comparison rumination. Scores on the SCRS were elevated among people with a general social comparison orientation and a propensity to make both upward and downward comparisons. Additional research confirmed links with other measures of rumination and automatic thoughts. We also identified extensive links with various ways of conceptualizing perfectionism and outcomes typically associated with perfectionism (e.g., burnout and distress). This work showed that there is a need to consider how people react to social comparison information and further investigate those students who find it difficult to mentally disengage from past and current comparisons. These people seem defined by a deleterious form of thinking and brooding that is linked with multiple costs and consequences.

## References

[bibr1-07342829241238300] Bandura A. (1971). Social learning theory. General Learning Press.

[bibr2-07342829241238300] Bandura A. Jourden F. J. (1991). Self-regulatory mechanisms governing the impact of social comparison on complex decision making. Journal of Personality and Social Psychology, 60(6), 941–951. 10.1037//0022-3514.60.6.941

[bibr3-07342829241238300] Burns D. D. (1980). The perfectionist’s script for self-defeat. Psychology Today, 3, 34–52.

[bibr4-07342829241238300] Butzer B. Kuiper N. A. (2006). Relationships between the frequency of social comparisons and self-concept clarity, intolerance of uncertainty, anxiety, and depression. Personality and Individual Differences, 41(1), 167–176.

[bibr5-07342829241238300] Buunk B. P. Ybema J. F. Gibbons F. X. Ipenburg M. (2001). The affective consequences of social comparison as related to professional burnout and social comparison orientation. European Journal of Social Psychology, 31(4), 337–351. 10.1002/ejsp.41

[bibr6-07342829241238300] Crowne D. P. Marlowe D. (1960). A new scale of social desirability independent of psychopathology. Journal of Consulting Psychology, 24(4), 349–354. 10.1037/h0047358 13813058

[bibr7-07342829241238300] De Lenne O. Vandenbosch L. Eggermont S. Karsay K. Trekels J. (2020). Picture-perfect lives on social media: A cross-national study on the role of media ideals in adolescent well-being. Media Psychology, 23(1), 52–78. 10.1080/15213269.2018.1554494

[bibr8-07342829241238300] Devos S. Schreurs L. Eggermont S. Vandenbosch L. (2023). Go big or go home: Examining the longitudinal relations between exposure to successful portrayals on social media and adolescents’ feelings of discrepancy. New Media and Society. 10.1177/14614448231188935

[bibr9-07342829241238300] Feinstein B. A. Hershenberg R. Bhatia V. Latack J. A. Meuwly N. Davila J. (2013). Negative social comparison on Facebook and depressive symptoms: Rumination as a mechanism. Psychology of Popular Media Culture, 2(3), 161–170. 10.1037/a0033111

[bibr10-07342829241238300] Festinger L. (1954). A theory of social comparison processes. Human Relations, 7(2), 117–140. 10.1177/001872675400700202

[bibr11-07342829241238300] Flett G. L. Hewitt P. L. (2022). Perfectionism in childhood and adolescence: A developmental approach. American Psychological Association. 10.1037/0000289-000

[bibr12-07342829241238300] Flett G. L. Hewitt P. L. Blankstein K. R. Gray L. (1998). Psychological distress and the frequency of perfectionistic thinking. Journal of Personality and Social Psychology, 75(5), 1363–1381. 10.1037//0022-3514.75.5.1363 9866193

[bibr13-07342829241238300] Flett G. L. Hewitt P. L. Davis R. A. Sherry S. B. (2004). Description and counseling of the perfectionistic procrastinator. In Schouwenburg H. C. Lay C. H. Pychyl T. A. Ferrari J. R. (Eds.), Counseling the procrastinator in academic settings. (pp. 181–194). American Psychological Association. 10.1037/10808-013

[bibr14-07342829241238300] Flett G. L. Hewitt P. L. Nepon T. Sherry S. B. Smith M. M. (2022). The destructiveness and public health significance of socially prescribed perfectionism: A review, analysis, and conceptual extension. Clinical Psychology Review, 93, 102130. 10.1016/j.cpr.2022.102130 35216826

[bibr15-07342829241238300] Flett G. L. Hewitt P. L. Whelan T. Martin T. R. (2007). The Perfectionism Cognitions Inventory: Psychometric properties and associations with distress and deficits in cognitive self-management. Journal of Rational-Emotive and Cognitive-Behavior Therapy, 25(4), 255–277. 10.1007/s10942-007-0055-4

[bibr16-07342829241238300] Flett G. L. Nepon T. Hewitt P. L. (2016). Perfectionism, worry, and rumination in health and mental health: A review and a conceptual framework for a cognitive theory of perfectionism. In Sirois F. M. Molnar D. S. (Eds.), Perfectionism, health, and well-being. (pp. 121–155). Springer International Publishing/Springer Nature. 10.1007/978-3-319-18582-8_6

[bibr17-07342829241238300] Flett G. L. Stainton M. Hewitt P. L. Sherry S. B. Lay C. (2012). Procrastination automatic thoughts as a personality construct: An analysis of the procrastinatory cognitions inventory. Journal of Rational-Emotive and Cognitive-Behavior Therapy, 30(4), 223–236. 10.1007/s10942-012-0150-z

[bibr18-07342829241238300] Gibbons F. X. Buunk B. P. (1999). Individual differences in social comparison: Development of a scale of social comparison orientation. Journal of Personality and Social Psychology, 76(1), 129–142. 10.1037//0022-3514.76.1.129 9972558

[bibr19-07342829241238300] Hewitt P. L. Flett G. L. (1991). Perfectionism in the self and social contexts: Conceptualization, assessment, and association with psychopathology. Journal of Personality and Social Psychology, 60(3), 456–470. 10.1037//0022-3514.60.3.456 2027080

[bibr20-07342829241238300] Hewitt P. L. Flett G. L. (2004). Multidimensional perfectionism scale: Technical manual. Multi-Health Systems Inc.

[bibr21-07342829241238300] Hewitt P. L. Flett G. L. Mikail S. F. (2017). Perfectionism: A relational approach to conceptualization, assessment, and treatment. Guilford Press.

[bibr22-07342829241238300] Hewitt P. L. Flett G. L. Sherry S. B. Habke M. Parkin M. Lam R. McMurtry B. Ediger E. Fairlie P. Stein M. B. (2003). The interpersonal expression of perfection: Perfectionistic self-presentation and psychological distress. Journal of Personality and Social Psychology, 84(6), 1303–1325. 10.1037/0022-3514.84.6.1303 12793591

[bibr23-07342829241238300] Ii A. Z. Sipps G. J. (1985). Cross-validation of a short form of the marlowe-crowne social desirability scale. Journal of Clinical Psychology, 41(2), 236–238. 10.1002/1097-4679(198503)41:2<236::aid-jclp2270410217>3.0.co;2-h

[bibr24-07342829241238300] Leary M. R. (1983). A brief version of the fear of negative evaluation scale. Personality and Social Psychology Bulletin, 9(3), 371–375. 10.1177/0146167283093007

[bibr25-07342829241238300] Lyubomirsky S. (2001). Why are some people happier than others? The role of cognitive and motivational processes in well-being. American Psychologist, 56(3), 239–249. 10.1037/0003-066X.56.3.239 11315250

[bibr26-07342829241238300] Molnar D. S. Blackburn M. Tacuri N. Zinga D. Flett G. L. Hewitt P. L. (2023). I need to be perfect or else the world’s gonna end”: A qualitative analysis of adolescent perfectionists’ expression and understanding of their perfectionism. In Canadian psychology. Advance online publication. 10.1037/cap0000357

[bibr27-07342829241238300] Nepon T. Flett G. L. Hewitt P. L. (2016). Self-image goals in trait perfectionism and perfectionistic self-presentation: Toward a broader understanding of the drives and motives of perfectionists. Self and Identity, 15(6), 683–706. 10.1080/15298868.2016.1197847

[bibr28-07342829241238300] Nolen-Hoeksema S. (2003). Women who think too much: How to break free of overthinking and reclaim your life. Holt.

[bibr29-07342829241238300] Nolen-Hoeksema S. Larson J. Grayson C. (1999). Explaining the gender difference in depressive symptoms. Journal of Personality and Social Psychology, 77(5), 1061–1072. 10.1037//0022-3514.77.5.1061 10573880

[bibr30-07342829241238300] Pincus A. L. Ansell E. B. Pimentel C. A. Cain N. M. Wright A. G. C. Levy K. N. (2009). Initial construction and validation of the pathological narcissism inventory. Psychological Assessment, 21(3), 365–379. 10.1037/a0016530 19719348

[bibr31-07342829241238300] Radloff L. S. (1977). The CES-D scale: A self-report depression scale for research in the general population. Applied Psychological Measurement, 1(3), 385–401. 10.1177/014662167700100306

[bibr32-07342829241238300] Reynolds W. M. (1982). Development of reliable and valid short forms of the marlowe-crowne social desirability scale. Journal of Clinical Psychology, 38(1), 119–125. 10.1002/1097-4679(198201)38:1<119::aid-jclp2270380118>3.0.co;2-i

[bibr33-07342829241238300] Rodebaugh T. L. Woods C. M. Thissen D. M. Heimberg R. G. Chambless D. L. Rapee R. M. (2004). More information from fewer questions: The factor structure and item properties of the Original and Brief Fear of Negative Evaluation Scale. Psychological Assessment, 16(2), 169–181. 10.1037/1040-3590.16.2.169 15222813

[bibr34-07342829241238300] Ruble D. N. Flett G. L. (1988). Conflicting goals in self-evaluative information-seeking: Developmental and ability level analyses. Child Development, 59(1), 97–106. 10.1111/j.1467-8624.1988.tb03198.x 3342717

[bibr35-07342829241238300] Sarason I. G. (1984). Stress, anxiety, and cognitive interference: Reactions to tests. Journal of Personality and Social Psychology, 46(4), 929–938. 10.1037/0022-3514.46.4.929 6737201

[bibr36-07342829241238300] Sarason I. G. Pierce G. R. Sarason B. R. (1996). Domains of cognitive interference. In Sarason I. G. Pierce G. R. Sarason B. R. (Eds.), Cognitive interference: Theories, methods, and findings (pp. 139–152). Lawrence Erlbaum Associates, Inc.

[bibr37-07342829241238300] Schaufeli W. B. Martinez I. M. Pinto A. M. Salanova M. Bakker A. B. (2002). Burnout and engagement in university students: A cross-national study. Journal of Cross-Cultural Psychology, 33(5), 464–481. 10.1177/0022022102033005003

[bibr38-07342829241238300] Schaufeli W. B. Salanova M. (2007). Efficacy or inefficacy, that’s the question: Burnout and work engagement, and their relationships with efficacy beliefs. Anxiety, Stress and Coping, 20(2), 177–196. 10.1080/10615800701217878 17999223

[bibr39-07342829241238300] Smith M. M. Saklofske D. H. Stoeber J. Sherry S. B. (2016). The big three perfectionism scale: A new measure of perfectionism. Journal of Psychoeducational Assessment, 34(7), 670–687. 10.1177/0734282916651539

[bibr40-07342829241238300] Smith M. M. Sherry S. B. Saklofske D. H. Mushqaush A. R. (2017). Clarifying the perfectionism-procrastination relationship using a 7-day, 14-occasion daily diary study. Personality and Individual Differences, 112(1), 117–123. 10.1016/j.paid.2017.02.059

[bibr41-07342829241238300] Smith R. H. Parrott W. G. Diener E. F. Hoyle R. H. Kim S. H. (1999). Dispositional envy. Personality and Social Psychology Bulletin, 25(8), 1007–1020. 10.1177/01461672992511008

[bibr42-07342829241238300] Stainton M. Lay C. H. Flett G. L. (2000). Trait procrastinators and behavior/trait-specific cognitions. Journal of Social Behavior and Personality, 15(5), 297–312.

[bibr43-07342829241238300] Treynor W. Gonzaelz R. Nolen-Hoeksema S. (2003). Rumination reconsidered: A psychometric analysis. Cognitive Therapy and Research, 27(3), 247–259. 10.1023/a:1023910315561

[bibr44-07342829241238300] van de Ven N. Zeelenberg M. (2020). Envy and social comparison. In Suls J. Collins R. L. Wheeler L. (Eds.), Social comparison, judgment, and behavior (pp. 226–250). Oxford University Press. 10.1093/oso/9780190629113.003.0009

[bibr45-07342829241238300] Vredenburg K. Flett G. L. Krames L. (1993). Analogue versus clinical depression: A critical reappraisal. Psychological Bulletin, 113(2), 327–344. 10.1037/0033-2909.113.2.327 8451338

[bibr46-07342829241238300] Weinstein E. (2017). Adolescents’ differential responses to social media browsing: Exploring causes and consequences for intervention. Computers in Human Behavior, 76, 396–405. 10.1016/j.chb.2017.07.038

[bibr47-07342829241238300] Wilson B. Gold T. (1991). Wouldn’t it be nice? In My own story. HarperCollins.

[bibr48-07342829241238300] Yang C. (2022). Social media social comparison and identity processing styles: Perceived social pressure to be responsive and rumination as mediators. Applied Developmental Science, 26(3), 504–515. 10.1080/10888691.2021.1894149

[bibr49-07342829241238300] Yang C. Robinson A. (2018). Not necessarily detrimental: Two social comparison orientations and their associations with social media use and college social adjustment. Computers in Human Behavior, 84(C), 49–57. 10.1016/j.chb.2018.02.020

